# Prevalence and spectrum of *MLH1*, *MSH2*, and *MSH6* pathogenic germline variants in Pakistani colorectal cancer patients

**DOI:** 10.1186/s13053-019-0128-2

**Published:** 2019-10-23

**Authors:** Muhammad Usman Rashid, Humaira Naeemi, Noor Muhammad, Asif Loya, Jan Lubiński, Anna Jakubowska, Muhammed Aasim Yusuf

**Affiliations:** 1 Department of Basic Sciences Research, Shaukat Khanum Memorial Cancer Hospital and Research Centre (SKMCH&RC), 7A, Block R3, Johar Town, Lahore, Punjab 54000 Pakistan; 2 Department of Pathology, SKMCH&RC, Lahore, Pakistan; 3 Department of Genetics and Pathology, Pomeranian Medical University, Szczecin, Poland; 4 Independent Laboratory of Molecular Biology and Genetic Diagnostics, Pomeranian Medical University, Szczecin, Poland; 5 Department of Internal Medicine, SKMCH&RC, Lahore, Pakistan

**Keywords:** HNPCC, Suspected-HNPCC, MMR genes, Pathogenic variants, Likely pathogenic variants, Pakistan

## Abstract

**Background:**

Pathogenic germline variants in *MLH1*, *MSH2* and *MSH6* genes account for the majority of Lynch syndrome (LS). In this first report from Pakistan, we investigated the prevalence of pathogenic *MLH1/MSH2/MSH6* variants in colorectal cancer (CRC) patients.

**Methods:**

Consecutive cases (*n* = 212) were recruited at the Shaukat Khanum Memorial Cancer Hospital and Research Centre (SKMCH&RC), between November 2007 to March 2011. Patients with a family history of > 3 or 2 HNPCC-associated cancers were classified as HNPCC (*n* = 9) or suspected-HNPCC (*n* = 20), respectively (group 1; *n* = 29). Cases with no family history were designated as non-HNPCC (group 2; *n* = 183). *MLH1/MSH2/MSH6* genes were comprehensively screened in group 1. Pathogenic/likely pathogenic variants identified in group 1 were subsequently evaluated in group 2.

**Results:**

Eight distinct pathogenic/likely pathogenic *MLH1/MSH2* variants were found in group 1 (10/29; 34.5%), belonging to HNPCC (5/9; 55.6%) and suspected-HNPCC (5/20; 25%) families and in group 2 (2/183; 1.1%) belonging to non-HNPCC. Overall, three recurrent variants (*MSH2* c.943-1G > C, *MLH1* c.1358dup and c.2041G > A) accounted for 58.3% (7/12) of all families harboring pathogenic/likely pathogenic *MLH1/MSH2* variants. Pathogenic *MSH6* variants were not detected.

**Conclusion:**

Pathogenic/likely pathogenic *MLH1/MSH2* variants account for a substantial proportion of CRC patients with HNPCC/suspected-HNPCC in Pakistan. Our findings suggest that HNPCC/suspected-HNPCC families should be tested for these recurrent variants prior to comprehensive gene screening in this population.

## Background

Colorectal cancer (CRC) is the fifth most common malignancy in Pakistan and endometrial cancer (EC) is the third most common gynecologic malignancy in Pakistani women [1]. The age-standardized (world) annual rates of CRC and EC are 4.0 and 3.6 per 100,000 in Pakistan, respectively. Affected individuals generally present at a young age. The majority of CRC and EC are not linked with inherited cancer syndromes. Up to 30% of CRC are hereditary and these may be divided into polyposis and non-polyposis syndromes. The term hereditary non-polyposis colorectal cancer (HNPCC) refers to patients and families who fulfill the Amsterdam criteria and differentiates familial aggregation of CRC from the polyposis phenotype. Up to 50% of HNPCC families have the Lynch syndrome (LS), with a DNA mismatch repair (MMR) defect, while the rest comprise those with a Lynch-like syndrome and a familial colorectal cancer type X (FCCTX) with no DNA MMR defects [2]. LS refers to families with a pathogenic germline variant in one of the DNA MMR genes (*MLH1, MSH2, MSH6,* and *PMS2*) or the *EPCAM* gene 3′ end deletions [3]. The most common pathogenic MMR gene variants (up to 90%) in LS are reported in *MLH1* and *MSH2* [4, 5], less commonly in *MSH6* (up to 10%) and uncommonly in *PMS2* [6]. Deletions in *EPCAM* gene (1–3%) in LS are rarely reported [7]. Individuals with LS have a lifetime risk of CRC, EC, and ovarian cancer ranging from 50 to 80%, 31.5–62%, and 6.7–13.5%, respectively. These individuals also face increased lifetime risks of developing cancer of the small bowel, stomach, upper urologic tract, biliary tract, pancreas and brain [8–12]. Identification of individuals harboring pathogenic MMR gene variants is clinically important and has a significant impact on surveillance and management [13].

Various clinical criteria such as the Amsterdam II criteria [14, 15] or the Bethesda guidelines exist for identifying patients at high risk of HNPCC. These criteria are based on a strong family history of at least three HNPCC-associated cancers, age at diagnosis and tumor histology. However, these stringent criteria have reported under-diagnosis of LS [16, 17]. Less stringent criteria of suspected-HNPCC, based on a family history of only two HNPCC-linked cancers, have also been found useful in identifying pathogenic variants in MMR genes [18–20].

The prevalence and spectrum of pathogenic MMR gene variants show considerable variation by ethnicity and by geographic origin worldwide [21–23]. However, little is known about the contribution of MMR gene variants to CRC in Pakistan. In the current study, we comprehensively investigated the contribution of pathogenic germline variants in *MLH1, MSH2* and *MSH6* genes to 212 Pakistani cases with HNPCC/suspected-HNPCC or non-HNPCC.

## Methods

### Study subjects

Consecutive cases were identified at the Shaukat Khanum Memorial Cancer Hospital and Research Centre (SKMCH&RC) in Lahore, Pakistan, from November 2007 to March 2011. These study cases were stratified into two groups: HNPCC/suspected-HNPCC group (*n* = 29) and non-HNPCC group (*n* = 183). Stringent criteria were applied for inclusion in the HNPCC subgroup. These included: (i) at least three relatives affected by histologically verified CRC or EC, small bowel or urinary tract; at least one of whom was a first degree relative of the other two, (ii) at least two of the above individuals were first degree relatives from two different generations, (iii) at least one of the above persons had cancer diagnosed at age under 50 years, (iv) familial adenomatous polyposis (FAP) had been excluded [14, 15]. Somewhat less stringent criteria used for the suspected-HNPCC subgroup included: (i) diagnosis of at least one CRC, EC, small bowel or urinary tract malignancy amongst first degree relatives of a CRC patient (or in him/herself), (ii) at least one of the above cancers diagnosed under age 50, (iii) FAP had been excluded [18]. The remaining 183 enrolled CRC cases did not fulfill the diagnostic criteria of HNPCC/suspected-HNPCC and were assigned to the non-HNPCC group. Clinical and histopathological data of all index patients were collected from medical records and pathology reports. A detailed description of the 212 index cases is shown in Table 1.

**Table 1:** Clinicopathological characteristics of HNPCC/suspected-HNPCC and non-HNPCC study participants

Characteristics	HNPCC/suspected-HNPCC (*n* = 27)^a^	non-HNPCC (n = 183)	*P* ^b^
Age at diagnosis of CRC (yrs)			
Mean	42.7	43.1	0.951^c^
Range	20–61	14–77	
< 50	21 (77.8)	115 (62.8)	0.194
> 50	6 (22.2)	68 (37.2)	
Gender, No (%)			
Male	21 (77.8)	125 (68.3)	0.377
Female	6 (22.2)	58 (31.7)	
Tumor location, No (%)			
Proximal	14 (58.3)	24 (13.2)	*** < 0.0001 ^**d**^
Distal	9 (37.5)	144 (79.6)	
Colon (not specified)	1 (4.2)	13 (7.2)	
Unknown	3	2	
Histologic type, No (%)			
Adenocarcinoma	20 (80.0)	142 (79.3)	1.0^e^
Mucinous adenocarcinoma	5 (20.0)	36 (20.1)	
Squamous cell carcinoma	0	1 (0.6)	
Unknown	2	4	
Mucinous component, No (%)			
Absent	20 (90.9)	132 (79.0)	0.257
Present	2 (9.1)	35 (21.0)	
Unknown	5	16	
Tumor size (cm), No (%)			
< 5	11 (45.8)	50 (70.4)	**0.047
> 5	13 (54.2)	21 (29.6)	
Unknown	3	112	
Macroscopic appearance, No (%)			
Ulcerative	5 (50.0)	17 (34.7)	0.061^f^
Infilterative	0	16 (32.7)	
Fungating	0	11 (22.4)	
Infiltrative+ulcerative	1 (10.0)	4 (8.2)	
Fungating+ulcerative	4 (40.0)	1 (2.0)	
Unknown	17	134	
Histologic grade, No (%)			
Low	18 (78.3)	99 (77.3)	1.0
High	5 (21.7)	29 (22.7)	
Unknown	4	55	
Lymphovascular invasion, No (%)			
Absent	16 (88.9)	32 (64.0)	0.197^g^
Present	2 (11.1)	14 (28.0)	
Intermediate	0	4 (8.0)	
Unknown	9	133	
Venous invasion, No (%)			
Absent	8 (100.0)	31 (79.5)	0.566^g^
Present	0	5 (12.8)	
Intermediate	0	3 (7.7)	
Unknown	19	144	
Primary tumor, No (%)			
pT0-pT2	8 (34.8)	17 (25.0)	0.421^h^
pT3	13 (56.5)	44 (64.7)	
pT4	2 (8.7)	7 (10.3)	
Unknown	4	115	
Regional lymph nodes, No (%)			
pN0	11 (47.8)	33 (50.0)	1.0^i^
pN1	7 (30.4)	15 (22.7)	
pN2	5 (21.7)	18 (27.3)	
Unknown	4	117	
Ethnicity, No (%)			
Punjabi	10 (37.0)	72 (39.3)	0.644^j^
Pathan	11 (40.8)	62 (33.9)	
Others	6 (22.2)	49 (26.8)	

*P* values marked in bold are statistically significant

*CRC* Colorectal cancer, *pN0* no regional lymph node metastasis, *pN1* metastasis in < 3 regional lymph nodes, *pN2* metastasis in > 4 regional lymph nodes, *pT2*, tumor invades through muscularis propria, *pT3* tumor invades through muscularis propria into pericolorectal tissues, *pT4* tumor directly invades other organs or structures

^a^ One index patient with breast-endometrial cancer and the other with ovarian cancer were not included

^b^ Fisher’s Exact test

^c^ Wilcoxon rank-sum test

^d^ Proximal vs. distal

^e^ Adenocarcinoma vs. mucinous adenocarcinoma

^f^ Ulcerative vs. infiltrative

^g^ Absent vs. present

^h^ pT0-pT2 vs. pT3–4

^i^ pN0 vs. pN1–2

^j^ Punjabi vs. Pathan

The control population included 100 healthy individuals of Pakistani origin, having no family history of CRC. These were care-givers or family members of hospital registered patients or those visiting the hospital for medical reasons other than cancer. All study participants were furnished with and signed an informed written consent. The study was approved by the Institutional Review Board (IRB) of the SKMCH&RC (IRB approval number SKMCH-CRC-001).

### Molecular analysis

Genomic DNA was extracted as previously described [24]. The entire coding region and exon-intron junctions of the *MLH1, MSH2* and *MSH6* genes (GenBank accession numbers NM_000249.3; NM_000251.2; NM_000179.2, respectively) were screened in 29 index patients of HNPCC/suspected-HNPCC group using denaturing high-performance liquid chromatography (DHPLC) analysis. The DHPLC analysis was carried out with the WAVE system (Transgenomics, Omaha, NE, US). PCR-primer pairs and DHPLC running conditions for *MLH1/MSH2* genes were according to Kurzawski and colleagues [4] and for *MSH6* gene was according to Kolodner et al. with some modifications [25] and are available upon request. When available, a positive control for each exon with a known variant was included in the DHPLC analyses.

Each sample showing variants detected by DHPLC analyses was sequenced using BigDye Terminator v.3.1 Cycle Sequencing Kit (Applied Biosystems, Foster City, CA, US), as described elsewhere [26]. Bidirectional genomic DNA sequencing was performed on an independent sample to verify the presence of a sequence variant.

Pathogenic/likely pathogenic variants identified in the HNPCC/suspected-HNPCC group were subsequently screened in the non-HNPCC group by DHPLC. Novel pathogenic variants and in silico predicted likely pathogenic variants were further analyzed in 100 healthy individuals.

### Classification of MMR gene variants

The MMR gene variants were stratified according to the following 5 tier classification, as described elsewhere: class 5 (pathogenic), class 4 (likely pathogenic), class 3 (uncertain significance), class 2 (likely benign) and class 1 (benign) [27]. The variants were designated as novel or previously reported variants by searching the following six databases: Exome Aggregation Consortium (ExAC), http://exac.broadinstitute.org/; Exome Sequence Project (ESP), http://evs.gs.washington.edu/EVS/; Human Gene Mutation Database (HGMD), http://www.hgmd.cf.ac.uk/ac/index.php; Leiden Open Variation Database (LOVD), https://databases.lovd.nl/shared/genes/; International Society for Gastrointestinal Hereditary Tumours (InSiGHT), https://insight-database.org/; Mismatch Repair Genes Variant Database (MMRGVD), http://www.med.mun.ca/mmrvariants/ or Universal Mutation Database (UMD), http://www.umd.be/ (by October 2016). The MMR gene variants identified in two or more unrelated patients were considered as recurrent variants.

### In silico analyses

The novel missense variants identified in *MLH1/MSH2* and previously reported class 3 variants of uncertain significance (VUS) in MMR genes were analyzed for their potential effect on protein function using the default settings of web tools Align-GVGD (http://agvgd.hci.utah.edu/agvgd_input.php), PolyPhen2 (http://genetics.bwh.harvard.edu/pph2/), SIFT (https://sift.bii.a-star.edu.sg/), Mut Pred (http://mutpred.mutdb.org/), SNPs&GO (http://snps.biofold.org/snps-and-go/snps-and-go.html), PhD SNP (http://snps.biofold.org/phd-snp/phd-snp.html), and SNAP (https://www.rostlab.org/services/snap/). Furthermore, all novel and previously reported intronic VUS in MMR genes were analyzed for their potential effect on splicing using the splice prediction algorithms SpliceSiteFinder-like (http://www.umd.be/searchSpliceSite.html), MaxEntScan (http://genes.mit.edu/burgelab/maxent/Xmaxentscan_scoreseq.html), NNSPLICE (http://www.fruitfly.org/seq_tools/splice.html), GeneSplicer (http://www.ccb.jhu.edu/software/genesplicer/) and HumanSpliceFinder (http://www.umd.be/HSF3/) via the Alamut software interface (Interactive Biosoftware) in default settings.

### Statistical analysis

The comparison of the distribution of clinical and histopathological characteristics between HNPCC/suspected-HNPCC group vs. non-HNPCC group and carriers of pathogenic/likely pathogenic *MLH1/MSH2* variant vs. non-carriers was performed using Fisher’s exact test for categorical variables and the Wilcoxon rank-sum test for quantitative variables. All statistical tests were two-sided. Results were deemed statistically significant if the *P* value was 0.05 or less. All statistical computations were done using StatXact 4 for Windows (Cytel Inc., Cambridge, US), SAS version 9.3 and R, version 2.1.

## Results

### Characteristics of the study participants

In total, 212 unrelated Pakistani index patients were included in the current study. Of these, 86.3% were diagnosed with CRC with no family history (non-HNPCC group = 183) and 13.7% reported a family history of cancer within the spectrum of HNPCC (HNPCC/suspected-HNPCC group = 29; 9 fulfilled the HNPCC criteria and 20 met the suspected-HNPCC criteria). Characteristics of the index CRC cases are shown in Table 1. Of the index cases, 210 patients including 146 males and 64 females had a diagnosis of CRC. Two patients belonged to the suspected-HNPCC subgroup: one with breast-endometrial cancer and the other with ovarian cancer. A majority of patients were of Punjabi (38.7%) or Pathan (34.4%) ethnic origin. The mean age at onset of disease was 42.7 years (range 20–61) and 43.1 years (range 14–77) for cases belonging to HNPCC/suspected-HNPCC group and non-HNPCC group, respectively (*P* = 0.95, Wilcoxon rank-sum test). The HNPCC/suspected-HNPCC group in comparison to non-HNPCC group more often presented with proximal tumor site (14/24, 58.3% vs. 24/181, 13.2%; *P* < 0.0001) and greater tumor size (> 5 cm) (13/24, 54.2% vs. 21/71, 29.6%; *P* = 0.047). There were no differences in histological type, mucinous component, macroscopic appearance, histologic grade, lymphovascular or venous invasion, tumor stage and lymph node involvement between both groups.

### Pathogenic germline variants: HNPCC/suspected-HNPCC group

The index patients of HNPCC/suspected-HNPCC group (*n* = 29) were entirely screened for germline *MLH1*, *MSH2* and *MSH6* variants using DHPLC followed by DNA sequence analyses. Seven distinct pathogenic/likely pathogenic *MLH1/MSH2* variants were identified in 10 cases (10/29; 34.5%) (Table 2). No pathogenic *MSH6* variant was found. Of the identified carriers of pathogenic/likely pathogenic variants, five carriers (50%) met the HNPCC criteria and five carriers (50%) met the suspected-HNPCC criteria (Table 3).

**Table 2:** *MLH1*, *MSH2* and *MSH6* germline variants in Pakistani study participants

Gene	Location	Nucleotide change^a^	Amino acid change	Variant type	SNP link^b^	Classification	Prevalence N (%)			Previously described
							HNPCC/suspected-HNPCC cases (*N* = 29)	non-HNPCC cases (*N* = 183)	Healthy controls (*N* = 100)	
*MLH1*										
	Exon 1	c.67delG	p.E23Kfs*13	Frameshift	–	P	1 (3.4)	0	–	Yes
	Exon 12	c.1358dup	p.T455Dfs*24	Frameshift	–	P	2 (6.9)	0	–	Yes
	Exon 15	c.1672G > T	p.E558*	Nonsense	–	P	1 (3.4)	0	–	Yes
	Exon 18	c.2041G > A	p.A681T	Missense	rs63750217	P	1 (3.4)	1 (0.6)	–	Yes
	Intron 1	c.116 + 3A > T	–	Intronic	–	LP^c^	0	1 (0.6)	0	No
	Exon 8	c.655A > G	p.I219V	Missense	rs1799977	B	2 (6.9)	–	**–**	Yes
	Exon 17	c.1913G > T	p.G638 L	Missense	–	B^c^	0	2 (1.1)	–	No
	Exon 17	c.1919C > T	p.P640L	Missense	–	LP^c^	6 (20.7)	2 (1.1)	**–**	Yes
	Exon 17	c.1959G > T	p.L653 L	Silent	rs1800146	B	0	1 (0.6)	**–**	Yes
	Intron 1	c.116 + 4C > A	–	Intronic	–	B^c^	0	1 (0.6)	–	No
	Intron 13	c.1558 + 14G > A	–	Intronic	rs41562513	B	1 (3.4)	–	**–**	Yes
	Intron 14	c.1668-19A > G	–	Intronic	rs9876116	B	8 (27.6)	55 (30.1)	**–**	Yes
	Intron 17	c.1990-26 T > C	–	Intronic	–	B^c^	0	2 (1.1)	–	No
*MSH2*										
	Exon 12	c.1861C > T	p.R621*	Nonsense	–	P	1 (3.4)	0	–	Yes
	Exon 16	c.2656G > T	p.E886*	Nonsense	–	P	1 (3.4)	0	0	Yes^d^
	Intron 5	c.943-1G > C	p.G315Ifs*12	Splice site	–	LP	3 (10.4)	0	–	Yes
	Exon 13	c.2120G > A	p.C707Y	Missense	–	LP^c^	1 (3.4)	2 (1.1)	2 (2)	No
	Exon 6	c.984C > T	p.A328A	Silent	–	LB	2 (6.9)	0	–	Yes
	Exon 6	c.944G > T	p.G315 V	Missense	rs202026056	B^c^	0	1 (0.6)	–	Yes
	Exon 6	c.965G > A	p.G322D	Missense	rs4987188	B	1 (3.4)	10 (5.5)	–	Yes
	Exon 6	c.1074G > C	p.E358D	Missense	–	B^c^	0	1 (0.6)	–	No
	Exon 12	c.1786_1788delAAT	p.N596del	In-frame deletion	–	P	0	1 (0.6)	–	Yes
	Exon 13	c.2205C > T	p.I735I	Silent	rs533553381	B	0	5 (2.7)	–	Yes
	Intron 1	c.211 + 9C > G	**–**	Intronic	rs2303426	LB	12 (41.4)	–	–	Yes
	Intron 9	c.1511-9A > T	–	Intronic	rs12998837	B	2 (6.9)	–	–	Yes
	Intron 10	c.1661 + 12G > A	–	Intronic	rs3732183	B	13 (44.8)	–	–	Yes
	Intron 12	c.2006-6 T > C	–	Intronic	rs2303428	B	1 (3.4)	37 (20.2)	–	Yes
	Intron 12	c.2006-36_2006-33dup	–	Intronic	rs587779126	B^c^	0	5 (2.7)	–	Yes
*MSH6*										
	Exon 3	c.540 T > C	p.D180D	Silent	rs1800935	B	1 (3.4)	–	**–**	Yes
	Exon 4A	c.642C > T	p.Y214Y	Silent	rs1800937	B	1 (3.4)	–	**–**	Yes
	Exon 4G	c.3151G > A	p.V1051I	Missense	–	B^c^	1 (3.4)	–	–	Yes
	Exon 5	c.3306 T > A	p.T1102 T	Silent	rs2020910	B	1 (3.4)	–	**–**	Yes
	Intron 2	c.457 + 13A > G	–	Intronic	rs1800933	LB	1 (3.4)	–	**–**	Yes
	Intron 2	c.457 + 50 T > A	–	Intronic	–	B^c^	3 (10.3)	–	–	No
	Intron 2	c.457 + 52 T > A	–	Intronic	rs3136282	B	23 (79.3)	–	**–**	Yes
	Intron 4	c.3172 + 20 T > C	–	Intronic	rs3136335	B	2 (6.9)	–	**–**	Yes
	Intron 5	c.3438 + 14A > T	–	Intronic	rs2020911	B	15 (51.7)	–	**–**	Yes
	Intron 6	c.3556 + 146G > A	–	Intronic	rs7562048	B	1 (3.4)	–	**–**	Yes
	Intron 6	c.3556 + 160 T > C	–	Intronic	rs56320267	B	1 (3.4)	–	–	Yes
	Intron 6	c.3556 + 170delT	–	Intronic	–	B^c^	1 (3.4)	–	–	No
	Intron 6	c.3557–4 dupT	–	Intronic	–	B	1 (3.4)	–	–	Yes
	Intron 6	c.3557-40 T > A	–	Intronic	rs189436849	LB	1 (3.4)	–	–	Yes
	Intron 9	c.4001 + 26A > G	–	Intronic	–	B^c^	1 (3.4)	–	–	No

*B* Benign, *LB* Likely benign, *LP* Likely pathogenic. *P* Pathogenic

^a^Nomenclature follows Human Genome Variation Society (HGVS) (http://www.hgvs.org). Numbering start at the first A of the first coding ATG of NCBI reference sequences

NM_000249.3(*MLH1*), NM_000251.2 (*MSH2*) and NM_000179.2 (*MSH6*)

^b^Link to NCBI SNP database (http://ncbi.nlm.nih.gov/projects/SNP/)

^c^Classification of the alterations is based on in silico analyses

^d^Previously reported in Pakistani population [28]

**Table 3:** Clinical criteria and frequencies of pathogenic *MLH1/MSH2* variants in Pakistani study participants

Clinical diagnostic criteria	N	with pathogenic variants N (%)	without pathogenic variants N (%)	*P* ^*a*^
HNPCC	9	5 (55.6)	4 (44.4)	*** < 0.0001^**b**^**,** 0.2047^c^
suspected-HNPCC	20	5 (25.0)	15 (75.0)	***0.0001 ^**d**^
non-HNPCC	183	2 (1.1)	181 (98.9)	
Total cases	212	12 (5.7)	200 (94.3)	

*P* values marked in bold are statistically significant

^a^Fisher’s exact test

^b^HNPCC vs. non-HNPCC

^c^HNPCC vs. suspected-HNPCC

^d^suspected-HNPCC vs. non-HNPCC

### *MLH1* variants

Five pathogenic variants (including four distinct variants) were detected in *MLH1* (5/29; 17.2%). Among these were two frame shift variants (including a recurrent variant), one nonsense variant and one missense variant (Table 4)*.*

**Table 4:** Characteristics of the families with pathogenic/likely pathogenic *MLH1/MSH2* variants

Study Id	Nucleotide change	Gender	Age at onset	Tumor location	Family history (age at onset in years)	Criteria	LOVD^a^	Ethnicity
Families with *MLH1* variants								
C162	c.1672G > T	F	32	Transverse colon	CRC (32, 45, 45,?,?), BC (42, > 45), unknown (?)	HNPCC	P	Kashmiri
C92	c.2041G > A	M	41	Transverse colon	CRC (42)	suspected-HNPCC	P	Punjabi
C122	c.2041G > A	M	41	Rectum	Brain tumor (16)	non-HNPCC		Urdu speaking
C203	c.1358dup	F	44	Sigmoid colon	CRC (< 30, 35, 54, 62), abdomen (?), stomach (36)	HNPCC	P	Punjabi
C202	c.67delG	F	48	Cecum	CRC (38, 42, 45)	HNPCC	P	Pathan
H707	c.1358dup	M	61	Transverse colon	CRC (31, 35, 45, 45, < 50, 50, 61,?)	HNPCC	P	Punjabi
Families with *MSH2* variants								
C143	c.943-1G > C	M	32	Rectosigmoid	CRC (40, 59, 60)	HNPCC	LP	Pathan
C164	c.1786_1788delAAT	M	39	Ascending colon	BC (50)	non-HNPCC	P	Punjabi
H1075	c.943-1G > C	M	43	Ascending colon	CRC (55), unknown (< 21,?)	suspected-HNPCC		Pathan
C85	c.1861C > T	M	45	Rectum	CRC (65)	suspected-HNPCC	P	Punjabi
H421	c.2656G > T	F	48, 67	Endometrium, breast	CRC (43, 55, 59), BC (58, 60, 66/76, 67), OC (43, 51, 57), ALL (5), endometrium (46, 52, 53), intestine (42, 45), stomach (59), liver (60), prostate (58), renal (58), brain (13), osteosarcoma (13)	suspected-HNPCC	P	Pathan
C49	c.943-1G > C	M	60	Sigmoid colon	CRC (50)	suspected-HNPCC		Pathan
Families with novel *MLH1/MSH2* variants								
C141	c.116 + 3A > T^b^	M	30	Sigmoid colon	–	non-HNPCC	NR/LP^b^	Punjabi
C199	c.2120G > A^c^	M	38	Rectum	CRC (40, 45, 50, 52, 65,?,?)	HNPCC	NR/LP^c^	Pathan
C75	c.2120G > A^c^	F	38	Recto sigmoid	Brain tumor (?)	non-HNPCC		Punjabi
P53	c.2120G > A^c^	F	54	Rectum	–	non-HNPCC		Punjabi
Families with a previously reported *MLH1* variant								
C198	c.1919C > T	M	35	Transverse colon	CRC (25, 30, 43, 66,?)	HNPCC	VUS/LP^d^	Pathan
C199	c.1919C > T	M	38	Rectum	CRC (40, 45, 50, 52, 65,?,?)	HNPCC		Pathan
C72	c.1919C > T	F	38	Transverse colon	Bladder (50), Bone (50)	suspected-HNPCC		Pathan
C55	c.1919C > T	M	38	Cecum	CRC (60)	suspected-HNPCC		Pathan
P02	c.1919C > T	M	45	Transverse colon	CRC (?,?,?)	HNPCC		Pathan
H708	c.1919C > T	M	51	Ascending colon	CRC (50, 65)	suspected-HNPCC		Pathan
P01	c.1919C > T	M	52	Transverse colon	CRC (?,?), Endometrium (?), Spleen (?)	non-HNPCC		Pathan
C185	c.1919C > T	F	60	Colon	Stomach (15), Epithilial (18)	non-HNPCC		Pathan

?, age at diagnosis is not known

*ALL* Acute lymphoid leukemia, *BC* Breast cancer, *CRC* Colorectal cancer, *LP* Likely pathogenic, *NR* No record in LOVD database, *OC* ovarian cancer, *P* pathogenic, *VUS* variant of uncertain significance

^a^Classification is based on Leiden Open Variation Database (LOVD) maintained by International Society for Gastrointestinal Hereditary Tumours (InSiGHT)

^b^This variant is considered as likely pathogenic by four of the five splice-site prediction algorithms

^c^This variant is considered as likely pathogenic by five of the seven protein function prediction algorithms

^d^This variant is reported as VUS in LOVD database and considered in the current study as likely pathogenic by seven of the seven protein function prediction algorithms combined with functional assay [29]

A recurrent frame shift variant in exon 12, c.1358dup (p.T455Dfs*24), was identified in two unrelated patients of Punjabi ethnicity. One patient presented with carcinoma of the sigmoid colon at 44 years of age (III:3, Fig. 1a). The other patient was diagnosed with carcinoma of the transverse colon at age 61 (III:18, Fig. 1b). Both reported a family history of HNPCC.

**Fig. 1:**
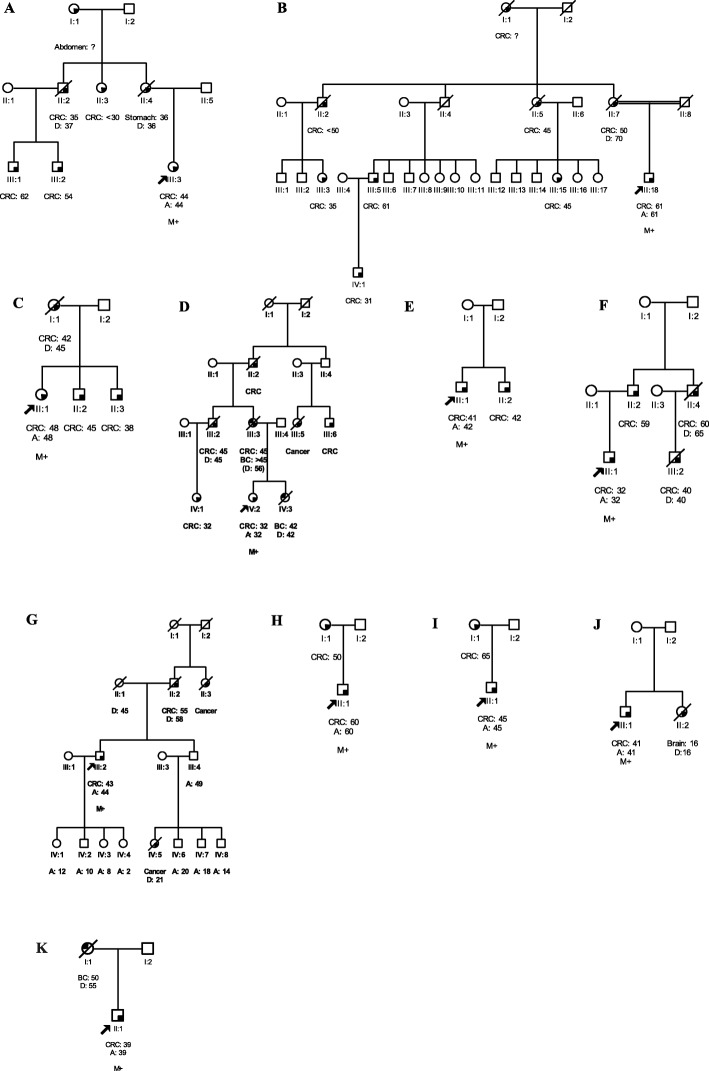
Pedigrees of HNPCC (**a-d** and **f**), suspected-HNPCC (**e** and **g-i**) and non-HNPCC (**j**, **k**) families with pathogenic/likely pathogenic *MLH1* or *MSH2* variants. **a-k**: Include families C203, H707, C202, C162, C92, C143, H1075, C49, C85, C122, and C164, respectively. Circles are females, squares are males, and a diagonal slash indicates a deceased individual. Symbols with filled left upper quadrant: unilateral breast cancer. Symbols with filled right lower quadrant: cancer other than breast, the name of that cancer is mentioned. Identification numbers of individuals are below the symbols. The index patient is indicated by an arrow. A*,* age; BC*,* breast cancer; CRC*,* colorectal cancer; D*,* death. The numbers following these abbreviations indicate age at enrollment, cancer diagnosis or death. M+, positive for pathogenic/likely pathogenic variant

Another frame shift variant in exon 1, c.67delG (p.E23Kfs*13), was detected in a 48-year-old patient (II:1, Fig. 1c) of Pathan ethnicity, who presented with carcinoma of the cecum and reported a family history of HNPCC.

A nonsense variant in exon 15, c.1672G > T (p.E558*), was identified in a 32-year-old patient (IV:2, Fig. 1d) of Kashmiri background, diagnosed with carcinoma of the transverse colon who also reported a family history of HNPCC.

One missense variant in exon 18, c.2041G > A (p.A681T), was identified in a 41-year-old patient (II:1, Fig. 1e) of Punjabi ethnicity with carcinoma of the transverse colon who reported a family history of suspected-HNPCC. This variant has been previously classified as a pathogenic variant [4, 30].

### *MSH2* variants

Five pathogenic/likely pathogenic variants (including three distinct variants) were identified in *MSH2* (5/29; 17.2%). Among these were one recurrent splice site variant and two nonsense variants (Table 4).

A recurrent likely pathogenic splice site variant, c.943-1G > C, was found in three unrelated patients of Pathan ethnicity: one with rectosigmoid carcinoma at 32 years of age (III:1, Fig. 1f) and a family history of HNPCC. The remaining two patients harboring this variant presented with carcinoma of the ascending colon (III:2, Fig. 1g) and sigmoid colon (II:1, Fig. 1h) at age 43 and 60, respectively and both reported a family history of suspected-HNPCC.

A pathogenic nonsense variant in exon 12, c.1861C > T (p. R621*), was identified in a 45-year-old patient (II:1, Fig. 1i) of Punjabi ethnicity, who was diagnosed with carcinoma of the rectum and also reported a family history of suspected-HNPCC.

Another pathogenic nonsense variant in exon 16, c.2656G > T (p.E886*), was identified in a 67-year-old patient of Pathan ethnicity, who was diagnosed with endometrial and breast cancer at age 48 and 67, respectively. This patient had a family history of suspected-HNPCC and has been reported recently [28].

### Pathogenic germline variants: non-HNPCC group

Screening of the index patients in the non-HNPCC group for the presence of the pathogenic/likely pathogenic *MLH1/MSH2* variants identified in the HNPCC/suspected-HNPCC group revealed two additional pathogenic *MLH1/MSH2* variants. The *MLH1* missense variant, c.2041G > A (p.A681T) was detected in a 41-year-old patient (II:1, Fig. 1j) of Urdu speaking background, who was diagnosed with carcinoma of the rectum. His sister (II:2, Fig. 1j) was diagnosed with a brain tumor (Table 4). The *MSH2*in-frame deletion (c.1786_1788delAAT) was identified in a 39-year-old CRC patient (II:1, Fig. 1k) of Punjabi ethnicity with a family history of breast cancer.

### Other MMR gene variants: novel or previously reported

In addition to the pathogenic/likely pathogenic variants, 35 distinct MMR variants including nine novel and 26 previously reported variants were detected. Among these were eight missense variants, six silent variants, and 21 intronic variants (Table 2).

The novel variants were analyzed for their potential functional effect by in silico analyses (Table 5). A novel *MLH1*splice-site variant, (c.116 + 3A > T), is predicted to be the likely pathogenic as suggested by four of the five splice-site prediction algorithms integrated into the Alamut software implying that this is disease-causative. This variant was identified in a 30-year-old patient of Punjabi origin, diagnosed with carcinoma of the sigmoid colon with no family history (Table 4). This variant was not found in 100 healthy controls, further supporting its pathogenicity.

**Table 5:** In silico analysis of the *MLH1, MSH2* and *MSH6* variants

Gene	Coding variants	In silico predictions							
		AlignGVGD	PolyPhen2	SIFT	MutPred	SNPs&GO	PhD-SNP	SNAP	Consensus^a^
*MLH1*	c.1913G > T (G638 L)	C15	Probably damaging	Deleterious	Benign	Neutral	Neutral	Neutral	B (3/7)
	c.1919C > T (P640L)	C65	Probably damaging	Deleterious	Deleterious	Disease	Disease	Disease	LP (7/7)
*MSH2*	c.944G > T (G315 V)	C0	Benign	Deleterious	Benign	Neutral	Neutral	Neutral	B (1/7)
	c.1074G > C (E358D)	C35	Possibly damaging	Tolerated	Benign	Neutral	Disease	Neutral	B (3/7)
	c.2120G > A (C707Y)	C0	Probably damaging	Damaging	Benign	Disease	Disease	Disease	LP (5/7)
*MSH6*	c.3151G > A (V1051I)	C0	Benign	Tolerated	Benign	Neutral	Neutral	Neutral	B (0/7)
Noncoding variants		Splice-site predictions							
		SpliceSiteFinder-like		MaxEntScan	NNSPLICE	GeneSplicer	HumanSpliceSite Finder		Consensus^b, c^
*MLH1*	c.116 + 3A > T	D (75.7 → 0)		D (8.6 → 2.4)	D (0.9 → 0)	D (5.5 → 0)	NE		LP (4/5)
	c.116 + 4C > A	NE		NE	NE	NE	NE		B (0/5)
	c.1990-26 T > C	NE		NE	NE	NE	NE		B (0/5)
*MSH2*	c.2006-36_2006-33dup	NE		NE	NE	NE	NE		B (0/5)
*MSH6*	c.457 + 50 T > A	NE		NE	NE	NE	NE		B (0/5)
	c.3556 + 170del	NE		NE	NE	NE	NE		B (0/5)
	c.4001 + 26A > G	NE		D (0 → 2.9)	NE	NE	D (0 → 71.4)		B (2/5)

*B* Benign, *D* Donor, *LP* Likely pathogenic, *NE* No effect

^a^The variant is considered as likely pathogenic by five of the seven protein function prediction algorithms

^b^ The variant is considered as likely pathogenic by four of the five splice-site prediction algorithms

^c^ > 20% change in score (i.e., a wild-type splice-site score decreases and/or a cryptic splice-site score increases) is considered as significant

A novel *MSH2* missense variant, c.2120G > A (p.C707Y)*,* is also predicted to be a likely pathogenic as suggested by five of the seven in silico prediction tools (Table 5). This variant was identified in three unrelated patients with CRC diagnosed at or below age 54: one patient of Pathan ethnicity reported a family history of HNPCC and two Punjabi patients of the non-HNPCC group (Table 4). Moreover, this variant was found in two out of 100 healthy controls including one with a family history of carcinoma of the pharynx and Ewing’s sarcoma. Characteristics of families harboring pathogenic/likely pathogenic *MLH1/MSH2* variants are shown in Table 4. The remaining seven novel MMR gene variants were also analyzed for their potential functional effect by in silico analyses and classified as benign.

Among the 26 previously reported MMR gene variants, 25 were benign or likely benign (Table 2). One *MLH1* missense variant, c.1919C > T (p.P640L), is predicted to be likely pathogenic as suggested by all seven in silico prediction tools used (Table 5). We identified this variant in eight unrelated CRC patients of Pathan ethnicity: six from the HNPCC/suspected-HNPCC group and two from the non-HNPCC group.

### Patient and tumor characteristics by variant status

The index CRC patients with pathogenic/likely pathogenic *MLH1/MSH2* variants (*n* = 11) and without pathogenic variants (*n* = 199) had a same median age of diagnosis, 43 years (range 32–61) and 43 years (range 14–77) of age, respectively (*P* = 0.74, Wilcoxon rank-sum test). The patients with pathogenic/likely pathogenic variants were more likely to present with proximal tumors (6/11, 54.5% vs. 26/194, 13.4%; *P* = 0.004) and greater tumor size (> 5 cm) (6/8, 75% vs. 28/87, 32.2%; *P* = 0.02) than non-carriers. No differences were detected between the carriers and non-carriers with regard to histologic type, mucinous component, macroscopic appearance, grade of malignancy, lymphovascular invasion, venous invasion, tumor stage, regional lymph node involvement and ethnic groups (data not shown).

## Discussion

In this first comprehensive study from Pakistan, we investigated the contribution of *MLH1, MSH2,* and *MSH6* pathogenic germline variants to 212 patients belonging to HNPCC/suspected-HNPCC group or non-HNPCC group. Initially, index patients from the HNPCC/suspected-HNPCC group (including HNPCC = 9 and suspected-HNPCC = 20; group 1) were screened for the entire coding sequence of these genes. The pathogenic/likely pathogenic variants identified in this group were then analyzed in the non-HNPCC group (*n* = 183; group 2). Eight different pathogenic/likely pathogenic variants in *MLH1*/*MSH2* were identified, with an overall frequency of 34.5% (10/29) in group 1 and 1.1% (2/183) in group 2. No pathogenic variants were detected in the *MSH6* gene. Among the group 1, five pathogenic *MLH1/MSH2* variants were detected in each subgroup of HNPCC and suspected-HNPCC, with frequencies of 55.6% (5/9) and 25% (5/20), respectively. The stringent criteria of HNPCC are two times more sensitive for detection of a pathogenic variant than the less stringent criteria of suspected-HNPCC. Our findings are in agreement with an international collaborative study reporting pathogenic variant detection rates of 50% (109/217) and 26% (32/123) for HNPCC and suspected-HNPCC criteria, respectively [20]. In our study, one in two patients identified with pathogenic variant did not meet the criteria of HNPCC, suggesting the need to use the criteria of suspected-HNPCC in Pakistani population.

Of the identified distinct pathogenic/likely pathogenic *MLH1/MSH2* variants (*n* = 8) in both groups, the *MSH2* variant, c.2656G > T, is likely to be specific to the Pakistani population as it has not been reported in other populations. The other seven variants have been reported in Asia, Europe, and North America [3, 30–37]. These findings suggest that the spectrum of *MLH1/MSH2* variants in Pakistan does not differ from other populations.

In the current study three distinct recurrent pathogenic/likely pathogenic variants in *MLH1* (*n* = 2) and *MSH2* (n = 1) were identified. The likely pathogenic *MSH2* variant, c.943-1G > C, was identified in three unrelated HNPCC/suspected-HNPCC families of Pathan ethnicity. It was also frequently reported in HNPCC families from Germany [33]. The pathogenic *MLH1* variant, c.1358dup, was found in two unrelated HNPCC families of Punjabi origin. This variant was recently found in HNPCC families from Australia [36]. The pathogenic *MLH1* variant, c.2041G > A, was detected in two unrelated suspected-HNPCC or non-HNPCC families of Punjabi and Urdu-speaking background, respectively. This variant was first reported in Poland as a potential founder variant [4, 31], has been reported as a recurrent variant in Scotland [30] and has also been described once each in Germany [33], and Colombia [3]. These recurrent variants accounted for 58.3% (7/12) of all *MLH1/MSH2* carriers from Pakistan. This further suggests a step-wise and cost-effective strategy of screening these recurrent variants, prior to the exhaustive analyses of MMR genes in our population. However, haplotype analysis of these recurrent variants is required to classify these as true Pakistani founder variants.

In addition to eight pathogenic/likely pathogenic variants found in twelve families, 35 MMR gene variants were detected: nine novel and 26 previously reported sequence variants*.* Of the novel sequence variants, two were suggested as in silico predicted likely pathogenic variants. The novel *MLH1*splice-site variant, c.116 + 3A > T, is predicted to be likely pathogenic as suggested by four of the five splice-site prediction algorithms. This variant was identified in a CRC patient of the non-HNPCC group and was not detected in 100 healthy controls. Further evidence of the impact of c.116 + 3A > T variant on aberrant mRNA splicing could not be provided because of the unavailability of an RNA sample from this patient. The novel *MSH2* missense variant, p.C707Y*,* is predicted to be likely pathogenic on the basis of the effect on protein function predicted by five of the seven in silico prediction tools. This variant was identified in three unrelated patients, one belonged to HNPCC group and other two were from the non-HNPCC group. It is located in the highly conserved ATPase domain (amino acid residues 620 to 855), may disrupt interaction of *MSH2* with other proteins in repair pathway and result in MMR defect [38]. This variant was detected in two out of 100 healthy controls with a family history of carcinoma of the pharynx or Ewing’s sarcoma. Functional analyses of both in silico predicted likely pathogenic novel variants (*MLH1* c.116 + 3A > T and *MSH2* p.C707Y) are warranted to further establish the association of these variants with the disease. One previously reported *MLH1* missense variant, p.P640L, is a likely pathogenic variant as predicted by seven in silico prediction tools used. This variant was identified in eight unrelated CRC patients of Pathan origin: six belonged to the HNPCC/suspected-HNPCC group while the other two were from the non-HNPCC group. This variant is located in a highly conserved C-terminal interaction domain (amino acid residues 492 to 756) and may ablate interaction of *MLH1* with *PMS2* and result in the MMR defect. Previously, Hardt and colleagues performed two functional assays and characterized p.P640L as a pathogenic variant [29]. Overall, these findings suggest that *MLH1* p.P640L is deemed to be a pathogenic variant.

In the current study, pathogenic/likely pathogenic *MLH1/MSH2* variants were identified in 34.5% (10/29) of Pakistani HNPCC/suspected-HNPCC patients, which is in agreement with other Asian studies from Korea (54/188; 28.7%), China (7/23; 30.4%), and Singapore (17/59; 28.8%) [39–41], Poland (78/226; 34.5%) [32], US (26/71; 36.6%) [42], and Brazil (44/116; 38%) [5]. A higher frequency of pathogenic variants was observed in HNPCC families from Taiwan (82/135; 60.7%) [43]. This could be due to screening of families who only met Amsterdam II or HNPCC criteria, whereas in this study we have also screened families who met the less stringent criteria of suspected-HNPCC. No pathogenic variant in *MSH6* was detected in the present study, in agreement with studies from China [44], and Singapore [40], suggesting a minimal contribution of *MSH6* variants in Asia. The predominance of pathogenic *MLH1*/*MSH2* variants and absence of *MSH6* variant in Pakistani population are in line with other ethnic mutation database [45]. These findings suggest that the contribution of pathogenic MMR gene variants to HNPCC/suspected-HNPCC families varies in Asians as well as in other populations.

Several criteria have been reported for the identification of potential candidates for the detection of pathogenic MMR gene variant. The most stringent and commonly applied Amsterdam II criteria [14, 15] is based on a family history of at least three relatives with histologically verified CRC or cancers linked with HNPCC. In our study, five out of nine patients belonging to families fulfilling this criterion were found to harbor a pathogenic *MLH1/MSH2* variant (5/9; 55.6%). The revised Bethesda guidelines recognize high-risk patients by the assessment of microsatellite instability and/or immunohistochemical testing of their tumors. However, this approach was not utilized due to limitations of normal/tumor tissue of study subjects. Nevertheless, the Amsterdam II criteria and Bethesda guidelines are shown to miss up to 72 and 27% of cases with HNPCC, respectively [17]. A recently suggested less stringent criteria of suspected-HNPCC are based on a family history of only two HNPCC-associated cancers [18–20]. In our study, five out of 20 patients belonging to families fulfilling this criterion were found to harbor a pathogenic *MLH1/MSH2* variant (5/20; 25%). Of the identified twelve carriers of pathogenic/likely pathogenic variant, five carriers met the HNPCC criteria and five met the suspected-HNPCC criteria and only two carriers were found in the non-HNPCC group. Our data support the notion that the suspected-HNPCC criteria may be useful for the identification of Pakistani families. The suspected-HNPCC criteria have also been utilized in other studies from Turkey, Poland, Italy and Latvia [31, 32, 37, 46].

In the current study, the frequency of pathogenic MMR gene variants observed in HNPCC/suspected-HNPCC group may be an underestimate as the sensitivity of DHPLC can be below 100% and screening for large genomic rearrangements or *EPCAM* gene 3′ end deletions was not performed. Furthermore, *PMS2* mutation screening was not performed. It is possible that we could have missed *PMS2* variants. However, pathogenic *PMS2* variants have only rarely been reported and accounted for less than 5% of all identified pathogenic MMR gene variants [7]. Finally, the contribution of additional undiscovered gene(s) in early onset CRC patients with a family history of LS-associated cancer who tested negative for any pathogenic MMR gene variants cannot be excluded. Thus, further studies in these patients are warranted.

Ethnic variations in frequencies of pathogenic *MLH1/MSH2* variant carriers have been reported in selected HNPCC families from Europe and US [21–23]. Similar ethnic variations in carrier frequencies of pathogenic/likely pathogenic *MLH1/MSH2* variants have been noted in our study. Of the identified variants, the majority of the families carrying *MLH1* variants (3/6; 50%) belonged to the Punjabi ethnicity. Majority of the families harboring pathogenic/likely pathogenic *MSH2* variants (4/5; 80%) had a Pathan background. These findings suggest that families with Punjabi or Pathan background should be first screened for the *MLH1* or *MSH2* gene, respectively. However, no firm conclusion could be made due to a small number of pathogenic *MLH1/MSH2* variant carriers. Furthermore, this study is not population-based and therefore might have some ascertainment bias.

Previous studies in Caucasians have predominantly reported the proximal tumor location in CRC patients harboring pathogenic MMR gene variants [47]. Similarly, in our study, CRC patients with pathogenic/likely pathogenic *MLH1/MSH2* variants more commonly presented with proximal tumor location compared to non-carriers. Similar observations have been noted in other Asian studies from Singapore [40], and Japan [48]. However, no such association was reported in studies from Korea [39] and China [49]. The differences in phenotypic manifestation may be due to ethnic variations or involvement of other genetic and/or non-genetic risk factors.

## Conclusion

In summary, this is the first comprehensive study conducted in Pakistani CRC patients to assess the prevalence and spectrum of *MLH1, MSH2,* and *MSH6* pathogenic germline variants. Pathogenic/likely pathogenic *MLH1/MSH2* variants account for a substantial proportion (10/29; 34.5%) of CRC patients with HNPCC/suspected-HNPCC in Pakistan, whereas no pathogenic *MSH6* variants were seen. Three recurrent *MLH1/MSH2* variants accounted for 58.3% (7/12) of all families carrying pathogenic/likely pathogenic variants. We recommend that HNPCC families, even those fulfilling the less stringent criteria of suspected-HNPCC, should first be tested for the recurrent pathogenic/likely pathogenic *MLH1/MSH2* variants prior to whole gene screening in Pakistani patients.

## Data Availability

All data generated or analyzed during this study are included in this published article.

## Abbreviations

CRC: Colorectal cancer
DHPLC: Denaturing high-performance liquid chromatography
EC: Endometrial cancer
*EPCAM*: Epithelial cell adhesion molecule
FAP: Familial adenomatous polyposis
FCCTX: Familial colorectal cancer type X
HNPCC: Hereditary non-polyposis colorectal cancer
IRB: Institutional Review Board
LS: Lynch syndrome
*MLH1*: MutL Homolog 1
*MMR*: Mismatch repair
*MSH2*: MutS Homolog 2
*MSH6*: MutS Homolog 6
*PMS2*: PMS1 Homolog 2
SKMCH&RC: Shaukat Khanum Memorial Cancer Hospital and Research Centre
